# Multiparametric cardiovascular magnetic resonance surveillance of acute cardiac allograft rejection and characterisation of transplantation-associated myocardial injury

**DOI:** 10.1186/1532-429X-16-S1-P394

**Published:** 2014-01-16

**Authors:** Christopher A Miller, Josephine H Naish, Steven M Shaw, Nizar Yonan, Simon G Williams, David Clark, Mark P Ainslie, Alex Borg, Glyn Coutts, Geoffrey J Parker, Simon G Ray, Matthias Schmitt

**Affiliations:** 1 North West Heart Centre and The Transplant Centre, University Hospital of South Manchester, Manchester, UK; 2 Centre for Imaging Sciences & Biomedical Imaging Institute, University of Manchester, Manchester, UK; 3 Institute of Cardiovascular Sciences, University of Manchester, Manchester, UK; 4 Alliance Medical Cardiac MRI Unit, University Hospital of South Manchester, Manchester, UK; 5 Christie Medical Physics and Engineering, The Christie Hospital, Manchester, UK

## Background

Acute cardiac allograft rejection (ACAR) affects approximately 20% of patients in the first year post-transplantation, represents a leading cause of death during this period and confers higher two- and four-year mortality in patients surviving beyond the first year. Clinical features of ACAR are unreliable, therefore serial surveillance endomyocardial biopsies are performed in order to detect ACAR at an early stage, however the biopsy process is associated with a number of limitations. This study aimed to prospectively and longitudinally evaluate the performance of multiparametric cardiovascular magnetic resonance (CMR) for detecting and monitoring ACAR in the early phase post-transplant, and characterize graft recovery following transplantation.

## Methods

All patients receiving a heart transplant at a single UK centre over a 25 month period of were prospectively approached within one month of transplantation. Multiparametric CMR was prospectively performed on the same day as biopsy on four separate occasions (to coincide with biopsies scheduled at 6 weeks, 10 weeks, 15 weeks and 20 weeks post-transplant). CMR included assessment of global and regional ventricular function (LV volumetrics, mass, ejection fraction, circumferential strain and strain rate, torsion (circumferential-longitudinal shear)), myocardial tissue characterisation (T1 mapping, T2 mapping, extracellular volume, LGE) and pixel-wise absolute myocardial blood flow quantification. CMR parameters were compared with biopsy findings. As is standard, > grade 2R ACAR was considered significant.

## Results

**Figure 1:**
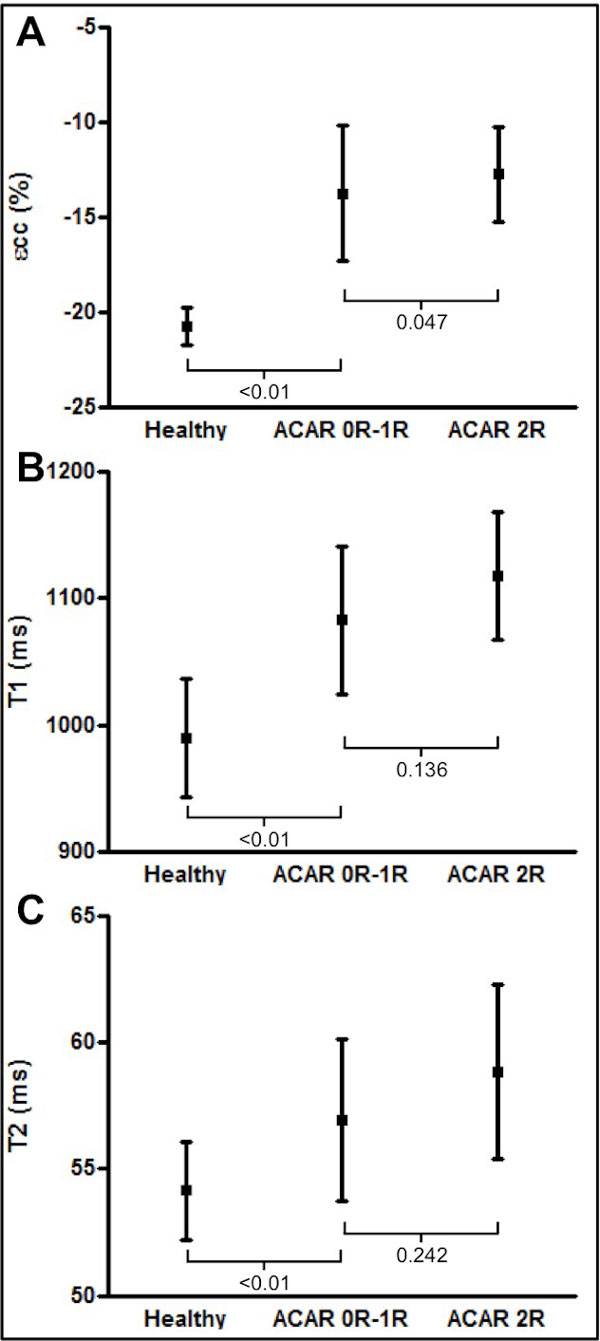
**Peak systolic circumferential strain (εcc, A), myocardial T1 relaxation time (B) and myocardial T2 relaxation time (C) in significant (grade 2R) and non-significant (grades 0R-1R) acute cardiac allograft rejection (ACAR) and in 10 matched healthy volunteers**.

**Figure 2:**
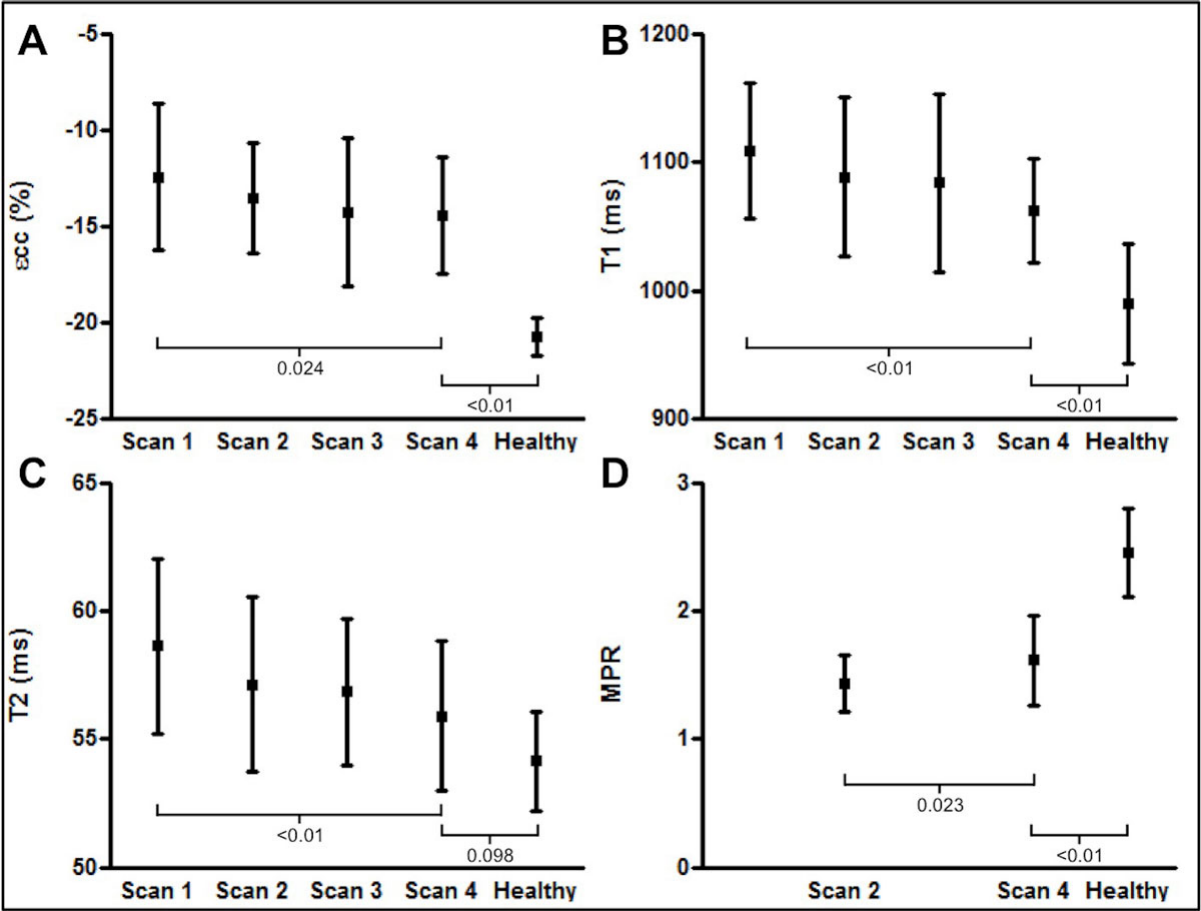
**Change in peak systolic circumferential strain (εcc, A), myocardial T1 relaxation time (B), myocardial T2 relaxation time (C) and myocardial perfusion reserve (MPR) over time from transplantation (scan 1: 6.9 weeks post-transplantation; scan 2: 10.9 weeks; scan 3: 16.6 weeks; scan 4: 22.3 weeks) and comparison of parameters at the time of scan 4 with 10 matched healthy volunteers**.

88 CMR-matched biopsies were performed in 22 patients. Eight (9%) biopsies in 5 patients demonstrated significant ACAR. Significant ACAR was associated with a reduction in circumferential strain (-12.7 ± 2.5% vs. -13.7 ± 3.6%, p = 0.047) but there was considerable overlap between groups (Figure 1). Whilst trends were observed between ACAR and CMR markers of oedema, differences were not significant (Figure 1). Significant improvements were seen in markers of graft structure and contractility, oedema and microvascular function over the period studied, although few parameters normalised (Figure 2). Primary graft dysfunction (a clinical syndrome defined as severe ventricular dysfunction developing immediately after transplantation and responsible for up to 40% of deaths in the early post-operative period), was associated with markedly abnormal markers of oedema and contractile function.

## Conclusions

This study provides novel insight into the myocardial injury associated with transplantation and primary graft dysfunction, and its recovery, however multiparametric CMR was not able to accurately detect ACAR during the early phase post-transplantation.

## Funding

CAM is supported by a Fellowship from the National Institute for Health Research, UK (NIHR-DRF-2010-03-98). CAM has received project funding from the Mason Medical Research Foundation Charity. CAM, SGW, NY and MS have received research funding from New Start Transplant Charity, UK.

